# *Nutritionally Variant Streptococci* Bacteremia in Cancer Patients: A Retrospective Study, 1999–2014

**DOI:** 10.4084/MJHID.2015.030

**Published:** 2015-04-20

**Authors:** Abraham T. Yacoub, Jayasree Krishnan, Ileana M. Acevedo, Joseph Halliday, John N. Greene

**Affiliations:** 1 H. Lee Moffitt Cancer Center and Research Institute. 12902 Magnolia Drive. Tampa, Florida 33612-9497.; 2 University of South Florida, Morsani College of Medicine, Division of Infectious Disease and International Medicine, 1 Tampa General Circle, G323.

## Abstract

**Background:**

*Nutritionally variant Streptococci (NVS), Abiotrophia* and *Granulicatella* are implicated in causing endocarditis and blood stream infections more frequently than other sites of infection. Neutropenia and mucositis are the most common predisposing factors for infection with other pathogens in cancer patients. In this study, we investigated the clinical characteristics of NVS bacteremia in cancer patients and identified risk factors and outcomes associated with these infections.

**Materials and Methods:**

We retrospectively reviewed all cases of NVS bacteremia occurring from June 1999 to April 2014 at H. Lee Moffitt Cancer Center and Research Institute. The computerized epidemiology report provided by the microbiology laboratory identified thirteen cancer patients with NVS bacteremia. We collected data regarding baseline demographics and clinical characteristics such as age, sex, underlying malignancy, neutropenic status, duration of neutropenia, treatment, and outcome.

**Results:**

Thirteen patients were identified with positive NVS blood stream infection. Ten patients (77%) had hematologic malignancies, including chronic lymphocytic leukemia (CLL)(1), multiple myeloma (MM)(1), acute myelogenous leukemia (AML)(4), and non-Hodgkin’s lymphoma (NHL)(4). The non-hematologic malignancies included esophageal cancer(2) and bladder cancer (1).

**Conclusion:**

NVS should be considered as a possible agent of bacteremia in cancer patients with neutropenia and a breach in oral, gastrointestinal and genitourinary mucosa (gingivitis/mucositis).

## Introduction

*Nutritionally Variant Streptococci (NVS)* (*Abiotrophia* and *Granulicatella*) are fastidious Gram-positive bacteria that were described for the first time in 1961.^1^ They are also called satelliting *streptococci* because they usually form satellite colonies around *Staphylococcus aureus* and other bacteria, including some *Enterobacteriaceae* and other *streptococci*.^2^ They are a common component of the oral flora but have been associated with a variety of invasive infections.^3^ Colonies of NVS are small (0.2 to 0.5 mm in diameter), and are either non-hemolytic or α-hemolytic on blood agar.^4^

The nutrient requirements of these microbes include cysteine or pyridoxal (active form of vitamin B6) for growth in complex media.^5^,^6^ NVS are divided in two genera (*Abiotrophia and Granulicatella*) comprising four species that have been identified from human specimens: *Abiotrophia defectiva, Granulicatella adiacens, Granulicatella elegans, and Granulicatella para-adiacens*.^2^

## Materials and Methods

We retrospectively reviewed all cases of NVS bacteremia occurring from June 1999 to April 2014 at H. Lee Moffitt Cancer Center and Research Institute. The computerized epidemiology report provided by the microbiology laboratory identified thirteen cancer patients with NVS bacteremia. We collected data regarding baseline demographics and clinical characteristics, such as age, sex, underlying malignancy, neutropenic status, duration of neutropenia, treatment, and outcome. Data was recorded from the Infectious Disease consultation reports and discharge summaries. Resolution of the infection was defined as repeated negative blood cultures. When reporting data, all percentages were rounded to the tenth decimal point.

## Results

**Figure 1:**
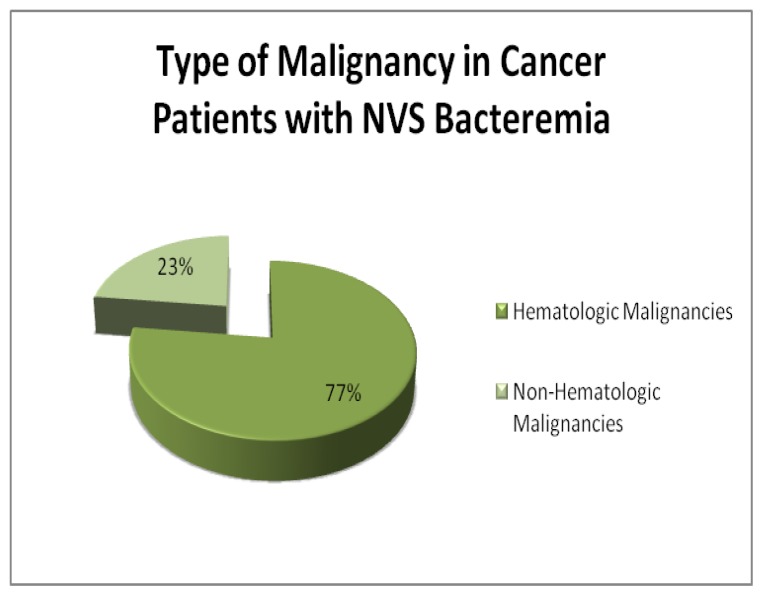
Type of Malignancy Associated with NVS Bacteremia

**Figure 2:**
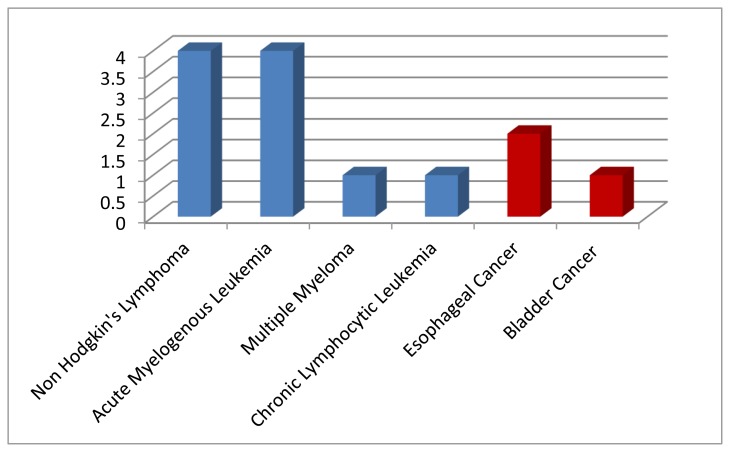
Hematologic vs Non Hematologic Malignancies

Thirteen patients were identified with positive NVS blood stream infection. Ten patients (77%) had hematologic malignancies, including chronic lymphocytic leukemia (CLL)(1), multiple myeloma (MM)(1), acute myelogenous leukemia (AML)(4), and non Hodgkin’s lymphoma (NHL)(4)(Table 1, Figures 1 and 2).

The non-hematologic malignancies included esophageal cancer(2) and bladder cancer(1). Seven patients (54%) were neutropenic (defined as Absolute Neutrophil Count < 1500 cells/uL) with an average duration of 14 days. The median age was 60 years. There was no gender predilection. Seven patients had mucositis at the time of diagnosis either due to chemotherapy or graft versus host disease. One patient had gingivitis with a dental abscess. None of the patients developed infective endocarditis. Most patients were on empiric antimicrobial therapy with ciprofloxacin, levofloxacin or piperacillin/tazobactam at the time of breakthrough bacteremia. Almost all patients received vancomycin as definitive treatment. All the patients had transient bacteremia with an average duration of positive blood cultures of 1 day. The 30-day mortality rate was 16.67%. Mortality was not attributable to NVS bacteremia

## Discussion

During this study, we collected the data spanning fourteen years period (1999–2014) at Moffitt Cancer Center. We found that NVS blood stream infections are commonly found in hematological malignancies. Our study was compared to Senn et al, and found that NVS blood stream infections were common in neutropenic patients with hematological malignancies.^1^

NVS are typically associated with endocarditis in immunocompetent patients and bacteremia in immunocompromised patients^1^,^7^ and interestingly none of our patients had developed endocarditis. Chemotherapy-induced mucositis and neutropenia have previously been identified as risk factors in cancer patients.^1^

NVS infections have been reported in patients with infectious crystalline keratopathy,^8^ vertebral osteomyelitis,^9^ endophthalmitis,^10^ meningitis^11^ and in cancer patients.^12^–^15^

Once a suspected NVS is cultured, its identity should be confirmed by establishing its requirement for pyridoxal.^2^ This test should be carried out on a medium that is incapable of supporting the organism’s growth without pyridoxal supplementation.^2^ A positive pyrrolidonyl arylamidase test along with typical morphology should further serve to identify an isolate as an NVS.^2^ The 16S rRNA gene PCR and restriction fragment length polymorphism analysis are different modalities to identify different species of NVS.^1^,^3^,^16^–^18^

Endocarditis caused by NVS has a higher rate of complications and treatment failure.^19^ NVS blood stream infections should be treated in the same way as viridans streptococci and enterococcus.^3^ It is recommended that a combination therapy of benzyl penicillin and amoxicillin plus a gentamicin for a course of 4 to 6 weeks is used to treat these microorganisms.^19^,^20^ Vancomycin is an alternative therapy when a penicillin-aminoglycoside combination is ineffective or contraindicated.^21^

In our study, most patients were on empiric antimicrobial therapy with ciprofloxacin, levofloxacin or piperacillin/tazobactam at the time of breakthrough bacteremia. Almost all patients received vancomycin as definitive treatment. All the patients had transient bacteremia with an average duration of positive blood cultures of 1 day.

Unlike streptococcus viridans, NVS does not typically cause adult respiratory distress syndrome and septic shock and is more benign.^22^,^23^ Although patients who develop fungemia, gram-negative bacteremia, or sepsis syndrome are best treated by catheter removal in addition to antimicrobial therapy, an increasing body of evidence suggests that many gram-positive bacterial catheter infections can be treated by use of antimicrobial agents without catheter removal.^24^,^25^

## Conclusion

NVS should be considered as a possible agent of Gram-positive bacteremia in cancer patients with neutropenia and a breach in oral or gastrointestinal mucosa, especially chemotherapy-induced mucositis or gingivitis. We recommend against routine removal of the central venous catheters given the benign course of NVS bacteremia, rapid clearance from blood, and likely oral or GI tract source of the pathogen. NVS bacteremia did not contribute to the mortality of patients in our study.

## Figures and Tables

**Table 1:** Nutritionally Variant Streptococci Bacteremia in Cancer Patients

No.	Age (years)	Gender	Malignancy	Duration of Bacteremia (Days)	Duration of Neutropenia (Days)	Treatment	Outcome
1	60	M	CML	1	-	Vancomycin, Levofloxacin	Survived
2	58	M	MM	1	-	Vancomycin, Cefepime	Deceased due to complications
3	76	M	AML	1	22	Vancomycin	Deceased due to complications
4	27	F	AML	1	34	Vancomycin, Tobramycin	Deceased at hospice
5	50	F	NHL	1	-	Bactrim	Deceased due to complications
6	64	F	NHL	1	7	Vancomycin, Cefepime	Survived
7	86	M	Bladder cancer	1	-	Ampicillin/sulbactam, Levofloxacin	Survived
8	39	F	NHL	1	4	Piperacillin/tazobactam	Deceased at hospice
9	59	M	Esophageal cancer	1	-	Piperacillin/tazobactam, Linezolid	Deceased due to complications
10	49	M	NHL	1	19	Cefepime, Vancomycin	Survived
11	66	M	Esophageal cancer	1	-	Aztreonam, Linezolid	Survived
12	66	M	AML	1	10	Piperacillin/tazobactam, Vancomycin	Survived
13	80	M	AML	1	2	Vancomycin	Survived

CML indicates Chronic myelogenous leukemia; MM, Multiple myeloma; AML, Acute myeloid leukemia; NHL, Non-Hodgkin lymphoma
